# Infantile Therapeutics

**Published:** 1894-12

**Authors:** 


INFANTILE THERAPEUTICS.—Luzet gives a critical review based on the work of Legendre and Broca. The special points really consist in the phases of development in the infant, in the special feature of the disease which here proceeds rapidly towards aggravation or recovery, and in the physiological peculiarities of more active metabolism, of more rapid absorption and circulation, of intact emunctories and of a more impressionable nervous system. In regard to feeding, the regular increase in weight must be relied upon. A tuberculous nurse must not be employed, for if bacilli do not pass out with the milk, toxins can; in addition, the milk is less rich in fat and casein. Overfeeding the nurse must be avoided. Of course, artificial feeding is only a method of necessity. The milk should be sterilized by means of steam under pressure. The therapeutic baths used to reduce temperature; the baths then gradually cooled down from two degrees F. below the child's temperature to 30 degrees F.; it is useful in enteric fever, severe scarlet fever and cerebral rheumatism. The bath with increasing temperature is of value in collapse such as occurs in diarrhœa; it may also be a vehicle for certain medicaments.

More strictly therapeutic measures are then discussed in the following order:

1. Evacuating medication. The stomach tube is very useful, as well as intestinal injections and emetics. Apomorphine is dangerous.

2. Promotion of excretions. The best diuretic is water. Large rectal injections of cold water constitute a good method of inducing diuresis. In uræma, icterus, and all intoxicatins large injections are useful. Cold baths are also of service in increasing renal excretion. Digitalis is well borne by children. Diaphoresis is best obtained by physical agents—heat, wet sheets, hot drinks. Diuresis is more efficient than diaphoresis.

3. Sleep should not be interrupted in disease, with very few exceptions. It may at times be necessary to induce sleep. This may sometimes be done by removing something which interferes with sleep. Physical agents are again the best means, such as tepid baths, etc. Opium requires caution; chloral is useful; bromides and antipyrin may be of service.

4. Fever is controlled by external agents—baths, etc. Quinine, antipyrin and sodium salicylate may be useful adjuvants.

5. Food is the best tonic. Alcohol is the best stimulant.

6. Antiseptic medication plays a very important part in infantile therapeutics. Carbolic acid in any form must be avoided. The mouth should be cleansed with alkaline lotions. Glycerine is a good non-fermentable medium. Antisepsis of the stomach may be procured by washing it out, and, together with the intestine by the use of bismuth, salicylate, salol, etc. Calomel is a powerful intestinal antiseptic. Antisepsis of the large intestine is obtained by means of irrigations containing naphthol, etc. It is indicated in typhlitis, appendicitis, membranous colitis, etc.—*British Med. Journal.*—*The Southern Clinic.*

